# Mad Dogs

**Published:** 1882-02

**Authors:** 


MONTHLY.

E. H. GIBBS, A. M., M. D., EDITOR.

FOUNDED IN 1854.

### MAD DOGS.

MUCH has been written about mad dogs, and the nature, symptoms and treatment of hydrophobia. Some physicians, eminent in their profession, have contended that there is no such disease, and have scouted at the idea that there was anything poisonous about the saliva or the teeth of a rabid dog; they attribute the so-called symptoms of hydrophobia to nervous anxiety, and say that the disease exists only in the imagination. There are well-authenticated cases of death resulting solely from fear, and such fear is quite as likely to result from the bite of a healthy dog as from any other cause. But to deny the existence of that terrible disease would be to assert that infants and the lower orders of animals may be tortured to death through the imagination, for these have been bitten and died in convulsions at a certain well-determined period after having been wounded by the teeth of rabid dogs; moreover, there are numerous instances where both children and adults have died of hydrophobia after having been bitten by dogs that manifested no signs of disease, and that have lived for years after causing the disease. I distinctly remember two cases of this kind. One was that of a young man in Brooklyn, who was slightly scratched on the nose by the teeth of a pet dog with which he was playing. He took but little notice of the wound, and, indeed, thought nothing about it until ten days later, when he was seized with symptoms of hydrophobia and died in great agony. I believe the dog was living several years afterward. Another similar case was that of McCormick, of New York, who was bitten by a dog just as he stepped into a meat market. McCormick died in a few days of hydrophobia; but the butcher refused to destroy his dog, and it was living two or three years afterward. There is no animal so loved and tenderly cared for as the dog; there is none other so deserving of such affection, or who returns it with such unchanging devotion; but this unfortunate liability to the most terrible of all diseases brings a protest from all prudent parents against his admission into the family. If there is one question about which parents and children differ, it is apt to be the dog question. The law supposes that every man is innocent until he is proved guilty; but I have reversed this rule as to dogs, and regard them as mad until they are dead and buried. It may seem unjust, but it is certainly safest, in view of what has happened frequently and what might at any time occur if we were to remit our caution. Should a dog bite me, I would treat myself without delay, without stopping to inquire if the dog was mad. All dogs that bite are mad dogs, and there is no safety in neglecting such wounds, and no peace of mind for years afterward to any person who knows the risk he is incurring. The poison from a dog bite does not enter the circulation so soon as that of a poisonous snake; there is time for more deliberation, but still it would be unwise to delay treatment unnecessarily. The first thing to be done is to get the poison out of the wound, and this is best done by sucking with the mouth, and squeezing the wound to expel the blood and the poison with it. Then the wound should be thoroughly washed with soap and warm water, and afterward touched thoroughly with nitric or carbolic acid. This is what the books say; but my plan of treating small poisonous wounds is more radical. After washing, I destroy with fire; not by the ancient method of the red-hot iron, but by a plan that is more certain and less painful. I insert the point of a common needle or pin into the wound and hold a lighted match to the eye of the needle or head of the pin till I have cooked the wounded part till it turns white, thus destroying all the contaminated flesh, and a little, perhaps, that is not contaminated, but I make a sure thing of it, regarding the permanent scar that will remain as a small matter in comparison to the safety of the operation.

---

THE longer I live the more deeply am I convinced that that which makes the difference between one man and another, between the weak and the powerful, the great and the insignificant, is energy, invincible determination ; a purpose once formed, and then death or victory. This quality will do anything that is to be done in the world, and no two-legged creature can become a man without it.—*Charles Buxton.*

